# Evidence against a Human Cell-Specific Role for LRP6 in Anthrax Toxin Entry

**DOI:** 10.1371/journal.pone.0001817

**Published:** 2008-03-19

**Authors:** Patricia L. Ryan, John A. T. Young

**Affiliations:** 1 Infectious Disease Laboratory, The Salk Institute for Biological Studies, La Jolla, California, United States of America; 2 Division of Biological Sciences, University of California San Diego, La Jolla, California, United States of America; The Research Institute for Children at Children's Hospital New Orleans, United States of America

## Abstract

The role of the cellular protein LRP6 in anthrax toxin entry is controversial. Previous studies showed that LRP6 was important for efficient intoxication of human M2182 prostate carcinoma cells but other studies performed with cells from gene-knockout mice demonstrated no role for either LRP6 or the related LRP5 protein in anthrax toxin entry. One possible explanation for this discrepancy is that LRP6 may be important for anthrax toxin entry into human, but not mouse, cells. To test this idea we have investigated the effect of knocking down LRP6 or LRP5 expression with siRNAs in human HeLa cells. We show here that efficient knockdown of either LRP6, LRP5, or both proteins has no influence on the kinetics of anthrax lethal toxin entry or MEK1 substrate cleavage in these cells. These data argue against a human-specific role for LRP6 in anthrax toxin entry and suggest instead that involvement of this protein may be restricted to certain cell types independently of their species of origin.

## Introduction

*Bacillus anthracis*, the etiological agent of anthrax, secretes a tripartite toxin, which is one of two major virulence factors. Anthrax toxin is composed of the receptor binding moiety, protective antigen (PA), and two catalytic moieties: Lethal factor (LF), a zinc –dependent metalloprotease that cleaves MAP kinase kinases (MAPKKs), [1]–[3] and edema factor (EF), a calcium- and calmodulin-dependent adenylate cyclase that raises cAMP levels [4]. LF and EF combine with PA to form lethal toxin (LeTx) and edema toxin (EdTx), respectively. Both 83 kD and 63 kD forms of PA (PA_83 _and PA_63_) can bind to either of two cellular receptors, ANTXR1 (anthrax toxin receptor 1/tumor endothelial marker 8; ATR/TEM8) or ANTXR2 (anthrax toxin receptor 2/capillary morphogenesis factor 2;CMG2) [5], [6]. Receptor-bound PA_83_ is cleaved by cell-surface furin into the 63 kD form [7]. PA_63_ forms a heptameric ring structure [PA_63(7)_], termed a prepore, at neutral pH [8]. EF and LF bind the prepore and the toxin/receptor complex is endocytosed primarily via clathrin-mediated endocytosis [9] and trafficked to an endocytic compartment where low-pH triggers PA_63(7)_ pore formation and translocation of EF and LF into the cytosol [10]–[12].

Currently there is a controversy about the role played in anthrax toxin entry by the low-density lipoprotein receptor-related protein LRP6, which interacts with both ANTXR1 and ANTXR2 [13]. Evidence in favor of a specific role was provided by Cohen and colleagues [13]. In that study, LRP6 was identified through a genome-wide antisense RNA screening approach to be important for intoxication of human M2182 prostate carcinoma cells by PA and FP59, a recombinant toxin comprised of the N-terminal portion of LF fused to *Pseudomonas* exotoxin A. Consistently, siRNA-mediated knockdown of LRP6 levels in these cells reduced their toxin sensitivity by several orders of magnitude, an effect that was partially overcome by expression of an siRNA-resistant form of LRP6. LRP6 deficiency in M2182 cells was also associated with reduced levels of PA binding and pore formation, and expression of a dominant-negative form of LRP6, lacking its cytoplasmic domain, rendered these cells resistant to intoxication. This group also showed that LRP6 could be co-precipitated with both ANTXR1 and ANTXR2. Furthermore, they showed that siRNA-mediated knockdown of LRP6 rendered RAW264.7 mouse macrophages resistant to intoxication by PA and LF, although this effect was much more modest (∼3-fold) than that seen with M2182 cells. LRP6-specific antibodies also protected RAW264.7 cells from intoxication.

By contrast, Duesbury and colleagues found that LRP6^+/−^ and LRP5^−/−^ mice were just as susceptible to killing after LeTx injection as wild-type mice [14]. LRP5 is a protein that is highly related to LRP6 [15]. In addition, they showed that mouse embryo fibroblasts (MEFs) that were isolated from LRP6^−/−^ or LRP5^−/−^ mice were just as susceptible to intoxication by PA and FP59, and to MEK1 cleavage by LF and PA, as those isolated from wild-type mice [14]. They went on to show that there is no obvious receptor-specific role for LRP6 since knocking down this protein had no effect on the toxin sensitivity of PA receptor-deficient Chinese hamster ovary cells that were engineered to express either ANTXR1 or ANTXR2.

Duesbury and colleagues put forward several possible explanations for the discrepant results. Since their data argue against an anthrax toxin receptor-specific role for LRP6, and there is no evidence for functional redundancy between LRP6 and LRP5 in toxin entry, they suggested that instead LRP6 might function in either a human-specific or cell type-specific manner. In this report we show that siRNA-mediated knockdown of LRP6 and/or LRP5 levels has no impact on the kinetics of anthrax toxin entry into human HeLa cells. These data argue against a human-specific role for either LRP6 or LRP5 in anthrax toxin entry and suggest that the requirement for LRP6 might be restricted to certain cell types.

## Results and Discussion

### Efficient siRNA-mediated knockdown of LRP6 and LRP5 expression in HeLa cells

**Figure 1:**
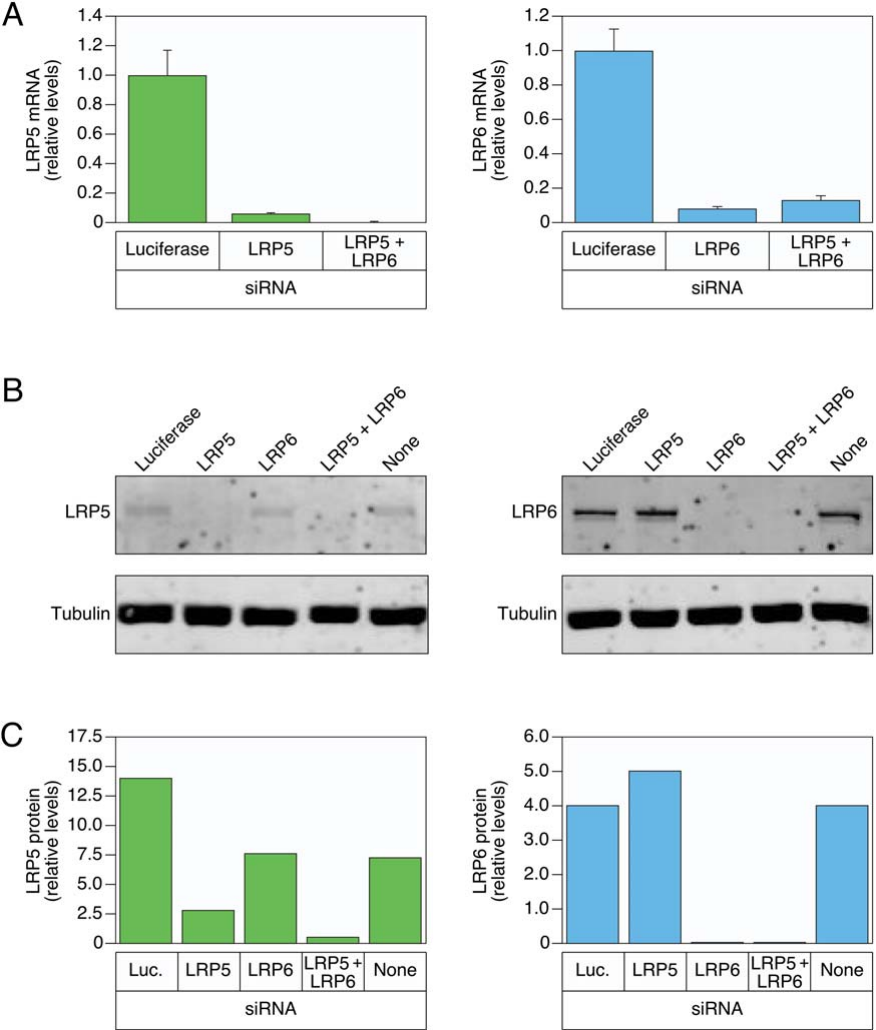
siRNA knockdown of LRP5 and LPR6 mRNA and protein expression in HeLa cells. A) RT-PCR analysis of LRP5 and LRP6 levels in HeLa cells transfected either with cognate pools of siRNAs or with siRNAs directed against luciferase (negative control). These experiments were each performed with triplicate samples and the mean average results are shown along with the standard deviation of the data indicated by error bars. B) Immunoblot analysis of LRP5 and LRP6 protein levels in cells transfected with the pools of siRNAs described in panel A. To control for equivalent cellular protein levels imunoblot analysis was also conducted on tubulin. C) The relative levels of LRP5 and LRP6 proteins in each sample shown in panel B were quantitated relative to the levels of tubulin in each sample using the fluorescence-scanning method described in materials and methods.

HeLa cells were chosen as a model human cell type because they have been used extensively for anthrax toxin entry studies and they are efficiently transfected with siRNAs [9], [16], [17]. To determine the efficiency of knocking down LRP6 or LRP5 expression, these cells were transfected with cognate siRNAs. These studies revealed an approximately 20-fold decrease in LRP5 and a 10-fold decrease in LRP6 mRNA transcripts in the transfected HeLa cells, relative to the levels observed with cells transfected with a negative control siRNA directed against luciferase (Fig. 1A). Consistently, siRNA transfection significantly reduced LRP5 and LRP6 proteins levels, as judged by immunoblotting (Fig. 1B and 1C). Importantly, LRP6 protein was reduced to undetectable levels in HeLa cells transfected with the corresponding siRNAs (Fig. 1B and C). Taken together these studies show that expression of LRP5 and LRP6 can be markedly reduced in siRNA-transfected HeLa cells. In addition, the level of siRNA-mediated knock-down of LRP5 and LRP6 exceeded those achieved in previous studies concerning the role of LRP5 and LRP6 in anthrax toxin internalization.

### LRP6 and LRP5 are not important for intoxication of HeLa cells

**Figure 2:**
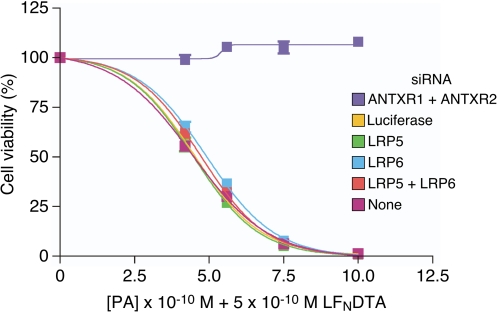
Reduced levels of LRP6 and/or LRP5 do not influence the toxin sensitivity of HeLa cells. siRNA-transfected cells were incubated with varying amounts of PA83 (as shown) and with 5×10^−10^ M LF_N_DTA. These experiments were performed with triplicate samples and the results shown are the mean average values with the standard deviation of the data indicated with error bars.

To test the importance of LRP6 and/or LRP5 for anthrax toxin entry, HeLa cells transfected with different siRNAs were tested for their susceptibility to intoxication in the presence of varied amounts of PA_83_ and a fixed amount of LF_N_-DTA. LF_N_-DTA is a hybrid toxin consisting of the PA-binding subunit of LF fused to the catalytic domain of diphtheria toxin A chain [18]. This recombinant toxin uses precisely the same PA-dependent pathway for cellular entry as that used by wild-type LF, and indeed it has been used as a convenient tool to study the anthrax toxin entry mechanism because, unlike wild-type LF, it can cause cell death in many cell types following its translocation into the cytosol [19]–[21]. For positive control purposes, HeLa cells were also transfected with siRNAs directed against both ANTXR1 and ANTXR2, which results in complete protection of the cells from intoxication because of efficient receptor knockdown (Fig. 2).

These studies revealed that the cells transfected with LRP5- and/or LRP6-directed siRNAs were just as susceptible to intoxication as were untransfected cells or cells transfected with a control firefly luciferase-specific siRNA (Fig. 2). These data argue against a specific requirement for either LRP6 or LRP5 in anthrax toxin entry.

### LRP6 and LRP5 do not influence the kinetics of PA_63(7)_ pore formation in HeLa cells

**Figure 3:**
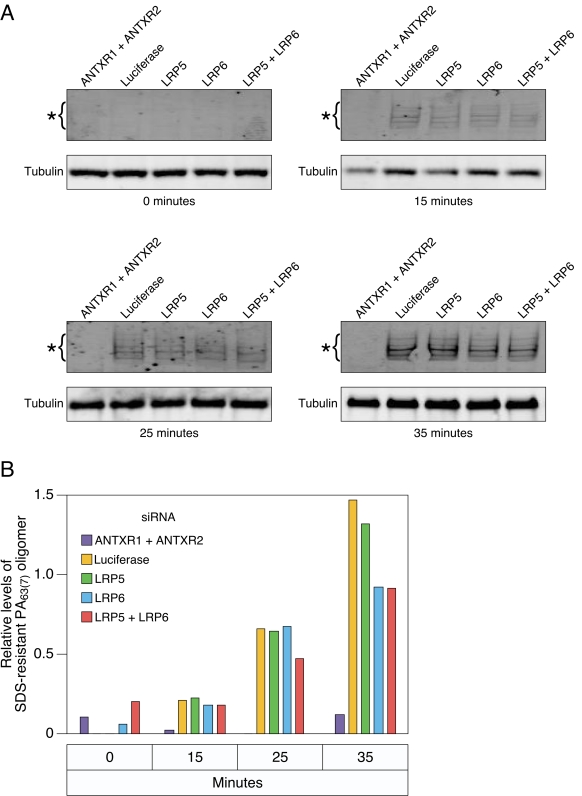
Reduced levels of LRP6 and/or LRP5 do not influence the kinetics of PA_63(7)_ pore formation in HeLa cells. A) The kinetics of PA pore formation in siRNA-transfected HeLa cells was evaluated as described in materials and methods. The cells were lysed at different time points after initiating toxin entry (0, 15, 25, 35 mins) and the protein lysates subjected to SDS-PAGE and immunoblotting with anti-PA antibody to detect the SDS-resistant oligomeric PA_63(7)_ pore species. (B) Quantitation of the levels of SDS-resistant pore species in the different time point samples shown in panel A. As described in materials and methods, the relative levels shown were determined by comparing the pore:tubulin ratio in each sample. The experiment shown is representative of three independent experiments.

The toxin sensitivity studies shown in Fig. 2 are conducted over a two day time frame and, as such, they do not provide any information on the kinetics of toxin entry which is usually complete within a 60- to 90-minute time period [2], [22], [23]. To explore the possibility that LRP6 and/or LRP5 might influence the kinetics of toxin entry we first evaluated the rate of PA63_(7)_ pore formation in siRNA-transfected HeLa cells. Pore formation was evaluated during the first 35 minutes after initiating toxin internalization, using a standard approach, namely by the acid- pH-dependent conversion of PA63_(7)_ to an SDS-resistant oligomeric species [8], [XPATH ERROR: unknown variable "start".], . These studies revealed that the kinetics of pore formation in cells transfected with LRP5 and/or LRP6 siRNAs were precisely the same as those seen in cells transfected with the control luciferase siRNA (Fig. 3A and 3B). As expected, HeLa cells treated with siRNAs targeting ANTXR1 and ANTXR2 showed little quantifiable pore formation (Fig. 3A and 3B). Taken together these studies demonstrate no obvious effect of knocking down LRP5 and/or LRP6 expression on the early kinetics of toxin uptake and acid pH-dependent PA pore formation.

### LRP6 and LRP5 do not influence the kinetics of MEK1 substrate cleavage in HeLa cells

**Figure 4:**
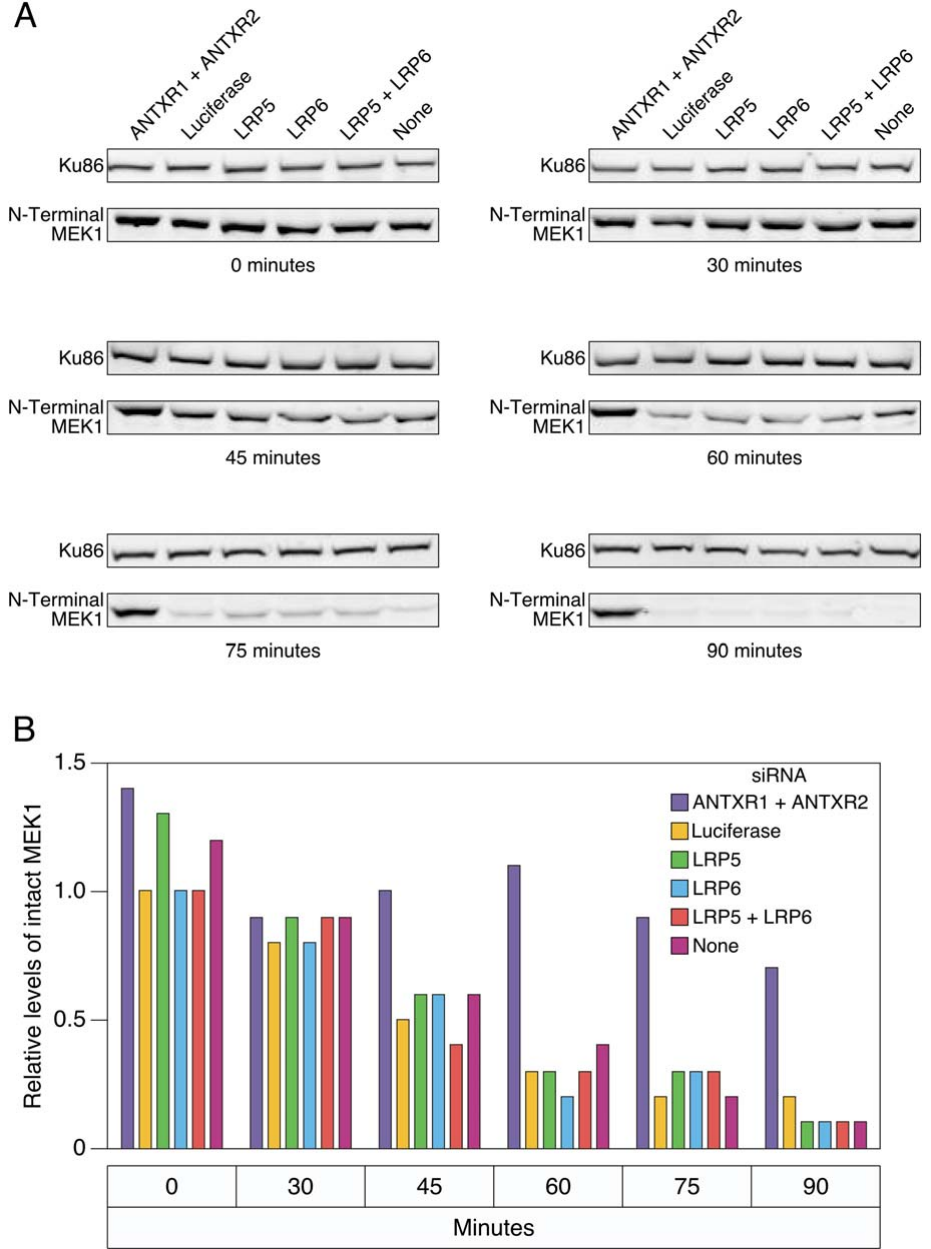
Reduced levels of LRP6 and/or LRP5 do not influence the kinetics of MEK1 cleavage in HeLa cells. A) HeLa cells transfected with different siRNAs were incubated with LeTx. Protein lysates were prepared at different time points and subjected to SDS-PAGE and immunoblotting with an anti-N-terminal MEK-1 antibody. Ku86, which is not an LF substrate, was also immunblotted as a loading control. B) The relative signal obtained with the N-terminal MEK1 antibody was compared with that associated with Ku86 in each sample shown in panel A. The experiment shown is representative of three independent experiments.

To confirm that LRP6 and LRP5 play no role in anthrax toxin entry into HeLa cells, we monitored the effect of knocking down expression of these proteins upon the kinetics of MEK1 cleavage by LF. LF cleavage removes the eight N-terminal amino acids of MEK1, an event that can be scored by immunoblotting using an antibody specific for the N-terminal region of MEK1 [1]–[3], [23]. MEK1 cleavage was monitored by immunoblotting at 0, 30, 45, 60, 75 and 90 minutes after LeTx addition and the signal obtained in each case was compared to that of Ku86, a cellular protein that is not a LF substrate. These studies revealed that siRNA-mediated knockdown of LRP6 or LRP5 levels had no influence on the MEK1 cleavage kinetics in HeLa cells (Compare these samples to the control samples transfected with luciferase siRNA: Fig. 4A and 4B). As expected, HeLa cells transfected with siRNAs targeting ANTXR1 and ANTXR2 were completely resistant to LF-mediated MEK1 cleavage (Fig. 4A and 4B).

In summary, the data obtained in this report argue against a human specific role for LRP6 or for the related LRP5 protein in anthrax intoxication. Efficient siRNA-mediated knockdown of expression of either or both LRP5 and LRP6 in HeLa cells had no impact on the kinetics of anthrax toxin uptake or pore formation in acidic endosomes. Furthermore, these treatments did not alter the kinetics of MEK1 cleavage following LeTx uptake into HeLa cells. These data lead us to conclude that LRP6 may be important for anthrax toxin uptake in different cell types independently of their species of origin, as was suggested by Duesbury and colleagues [14]. However, future studies in this area will be required to clarify the precise context(s) under which anthrax toxin entry is dependent upon LRP6.

## Materials and Methods

### siRNA transfections

HeLa cells were reverse-transfected using RNAiMax (Invitrogen) according to manufacturer's instructions with 32 nM siRNA. siRNAs were obtained from Dharmacon: LRP6 On-Target Plus SmartPool siRNA (GCAGAUAUCAGACGAAUUUUU, CAGAUGAACUGGAUUGUUAUU, CCACAGAGCGAUCACAUUAUU, GCUCAACCGUGAAGUUAUAUU) LRP5 On-Target Plus Smartpool siRNA (CGUCAAAGCCAUCGACUAUUU, CGUCAUGGGUGGUGUCUAUUU, GGACGGACCUACGGAGGAUUU, GUACAGGCCCUACAUCAUUUUU), ANTXR1 On Target SmartPool siRNA (CCAGUGAGCAGAUUUAUUAUU, GCUAAUAGGUCUCGAGAUCUU, GAAGAAGUCCUGCAUCGAAUU, GGAACAACCUUAAUGAAACUU) ANTXR2 On Target SmartPool siRNA (Dharmacon GUAAAGGCUUGGAGGAUUUU, GCUAGCGAAUGAACAAAUUUU, GGGCUAGUGUUUAUUGUGUUU, UAUACUAGCUCAGUCAUGUUU) and GL2 firefly luciferase siRNA (target DNA sequence: CGTACGCGGAATACT TCGA)

### LRP5 and LRP6 protein expression analysis

Approximately 5×10^4^ HeLa cells plated in individual wells of a 12-well plate were reverse-transfected with different siRNA pools targeting LRP5, LRP6, both LRP5 and LRP6, or firefly luciferase (negative control). After 48 hours, cells were lysed with reducing gel sample buffer containing 2% SDS and 100 mM DTT [24]. Lysate samples were separated by denaturing SDS-PAGE and transferred to PVDF membranes (Millipore). These membranes were blocked and incubated with antibodies in TBS-T containing 5% milk: The same conditions were used for all immunoblotting experiments described in this report. The antibodies used were the LRP5-specific (Rabbit anti-LRP5, Zymed) diluted 1∶200 and the LRP6-specific (LRP6 C5C7), Cell Signaling Technologies) diluted 1∶1000. Tubulin was detected as a loading control (α/β-Tubulin antibody, Cell Signaling Technologies) diluted 1∶1000. The Alexa Fluor 680-conjugated secondary antibody used in each case was the goat anti-rabbit IgG (Alexa Flour 680 goat anti-rabbit IgG, Invitrogen) diluted 1∶20,000. The samples were then scanned and analyzed with the fluorescence-scanning Odyssey system and its associated software (Li-Cor).

### RT-PCR analysis of LRP5 and LRP6 mRNA expression

Approximately 1×10^5^ HeLa cells plated in individual wells of a 6-well plate were reverse-transfected with the different siRNA pools described above. RNA was harvested using the Qiagen RNeasy kit at 48 hours post siRNA transfection for RT-PCR analysis: The cDNA synthesis was performed with the SuperScript III reverse transcriptase system (Invitrogen) using 2 µg of total RNA from each sample and random hexamers as primers. PCR amplification was subsequently performed for 40 cycles (95°C for 15 seconds, 60°C for 1 minute) with an ABI Prism 7900HT instrument and with primers obtained from the validated library of Qiagen Quantitect primers along with Sybr green PCR master mix (Applied Biosystems). The relative levels of LRP5 or LRP6 mRNA transcripts were then determined using the Comparative Ct (ΔΔCt) method using GAPDH (CCTCTGACTTCAACAGCGACAC, TTCCTCTTGTGCTCTTGCTGG) as an endogenous mRNA control.

### Cell intoxication assays

Triplicate samples of approximately 5×10^3^ HeLa cells seeded in individual wells of a 96-well plate were reverse-transfected with the different siRNAs. After 48 hours, cells were incubated with 5×10^−10^M LF_N_-DTA and with varying concentrations of PA for 48 hours [18]. Cell viability was then assayed with the Cell Titer-Glo assay (Promega) as previously described [24].

### SDS-resistant pore formation

Formation of the SDS-resistant PA_63(7)_ pore species was detected by immunoblotting as previously described [24], except cells were incubated with 2.5×10^−8^M PA and 2×10^−9^M LF for 2 hours at 4°C, washed and shifted to 37°C for the indicated times. Cells were lysed with reducing gel sample buffer, protein lysates were subjected to SDS-PAGE and SDS-resistant pore was detected with anti-PA (Goat anti-PA, List Labs) diluted 1∶2,000. The secondary antibody used in this case was the Alexa Fluor-680 conjugated rabbit anti-goat (Invitrogen) diluted 1∶20,000. The immunoblotting conditions were the same as described above and western blot scanning and analysis was conducted using the Odyssey system and its associated software (LiCor).

### MEK1 Cleavage assays

MEK1 cleavage was detected by immunoblotting as previously described [24], except that samples were collected directly with reducing gel sample buffer. Membrane blocking and antibody dilutions were performed with TBS-T containing 5% milk as described above. The N-terminal MEK1 antibody (Anti-MEK1 N-terminal) was obtained from Calbiochem (used at 1∶1,000) and the Ku86 antibody (Ku-86 B-1) was obtained from Santa Cruz Biotechnology (used at 1∶500 dilution). Alexa Fluor-680 conjugated anti-Rabbit IgG and Alexa Fluor-680 conjugated anti-mouse IgG (Invitrogen) were used as secondary antibodies (both diluted 1∶20,000). Western blot scanning and analysis was conducted using the Odyssey system and its associated software (LiCor).
